# Does Regular Exercise Counter T Cell Immunosenescence Reducing the Risk of Developing Cancer and Promoting Successful Treatment of Malignancies?

**DOI:** 10.1155/2017/4234765

**Published:** 2017-07-02

**Authors:** James E. Turner, Patricia C. Brum

**Affiliations:** ^1^Department for Health, University of Bath, Claverton Down, Bath BA2 7AY, UK; ^2^School of Physical Education and Sport, University of São Paulo, Av. Prof. Mello de Morais, 65 - Cidade Universitária, 05508-030 São Paulo, SP, Brazil

## Abstract

Moderate intensity aerobic exercise training or regular physical activity is beneficial for immune function. For example, some evidence shows that individuals with an active lifestyle exhibit stronger immune responses to vaccination compared to those who are inactive. Encouragingly, poor vaccine responses, which are characteristic of an ageing immune system, can be improved by single or repeated bouts of exercise. In addition, exercise-induced lymphocytosis, and the subsequent lymphocytopenia, is thought to facilitate immune surveillance, whereby lymphocytes search tissues for antigens derived from viruses, bacteria, or malignant transformation. Aerobic exercise training is anti-inflammatory and is linked to lower morbidity and mortality from diseases with infectious, immunological, and inflammatory aetiologies, including cancer. These observations have led to the view that aerobic exercise training might counter the age-associated decline in immune function, referred to as immunosenescence. This article summarises the aspects of immune function that are sensitive to exercise-induced change, highlighting the observations which have stimulated the idea that aerobic exercise training could prevent, limit, or delay immunosenescence, perhaps even restoring aged immune profiles. These potential exercise-induced anti-immunosenescence effects might contribute to the mechanisms by which active lifestyles reduce the risk of developing cancer and perhaps benefit patients undergoing cancer therapy.

## 1. Introduction and Overview

Since the first observations of the immune system being modulated by bouts of vigorous exercise in the 1890s, a substantial body of evidence has accumulated showing that most aspects of immune function are sensitive to exercise-induced change [1]. Many of these changes have been interpreted as being beneficial, and this view has led to aerobic exercise training being advocated as a method to prevent, limit, or delay the age-associated decline in immune function referred to as immunosenescence [2–4]. It has even been suggested that aerobic exercise training might rejuvenate aged immune profiles [2, 3], which is attractive given our ageing population, and the potential implications that immunosenescence has for increasing susceptibility to infections and increasing the risk of developing cancer.

The purpose of this article is to summarise the effects that aerobic exercise training and physical activity can have on aspects of immune function with an emphasis on how some of these changes could be considered as countering immunosenescence. Few studies have investigated changes to immune function brought about resistance exercise (e.g., lifting weights) or high-intensity sprint interval exercise; therefore, these activities will remain largely undiscussed. In addition, this article will summarise studies that have investigated whether immunosenescence influences the risk of developing cancer or affects the treatment of patients with a cancer diagnosis. Readers are directed towards articles that have reviewed these concepts in more depth, including the effects of exercise on immune function [1, 5, 6], the influence of chronological age and infection history on immunosenescence [7–9], the effects of aerobic exercise training on immunosenescence [2–4, 10], and the links between immunosenescence and cancer risk or treatment [11–13].

## 2. Characteristics of an Ageing Immune System

The profile of the innate and adaptive immune system is markedly different between young and elderly individuals. For example, with ageing, there is an increase in systemic inflammatory activity referred to as inflammageing [14, 15]. Age-associated alterations are also observed with the number, phenotype, and function of innate immune cells [9]. Neutrophils exhibit diminished phagocytosis and have an impaired capacity to control their direction of migration (chemotaxis), but their speed of movement (chemokinesis) is maintained. Mast cells decline in number but not function, whereas the number of eosinophils and basophils, along with their function, remains largely unchanged. Monocyte numbers in peripheral blood are stable with ageing, but classical cells (CD14++CD16−) decline, and intermediate (CD14+CD16+) and nonclassical (CD14+CD16++) cells increase [9, 16]. These numerical changes coincide with altered signalling via some toll-like receptors resulting in impaired cytokine production. Changes in monocytes due to age per se are thought to be mirrored by the phenotype and functional properties of tissue macrophages, whereby classically activated M1 cells decline and alternatively activated M2 cells accumulate [17]. However, the composition of tissue-resident cells is complicated by adipose tissue accumulation and dysfunction—dominated by M1 macrophages [18, 19]—and the M1/M2 paradigm is likely to be an oversimplification [20, 21]. It is unclear if ageing affects the number of dendritic cells in peripheral blood, but numbers decline in the skin and mucosal membranes, and antigen processing and presentation, cytokine production, costimulatory capacity, and migration are impaired. The number of natural killer cells increases with ageing, but this change varies by subtype; cytotoxic cells accumulate and regulatory cells decline; however, overall cytokine production and cytotoxicity are less on a per cell basis. With ageing, there is a decline in the number of invariant natural killer T cells (iNKT cells)—innate lymphocytes, which represent <1% of the T cell pool and recognise tumours or infected cells via CD1d-presented glycolipids [22]. It is possible that some of these age-associated changes are driven by an increase in myeloid-derived suppressor cells (MDSCs)—a heterogenous population of granulocytes, macrophages, and dendritic cells that suppress aspects of immune function by producing reactive oxygen species and inhibitory cytokines [23].

Within the adaptive immune system, a classical hallmark of ageing that predicts mortality in the elderly is impaired mitogen-induced T cell proliferation [24]. Many other age-associated changes to T cell numbers and phenotypes have been reported. For example, among T cell subpopulations, such as CD4+ T-helper (Th) cells, ageing is associated with a predominance of cells with a Th2 cytokine profile (i.e., IL-4 and IL-10 producing cells), whereas there is a decline in cells with a Th1 profile (i.e., IFN-*γ* and TNF-*α* producing cells) [25]. Another functional shift in T-helper cell profile includes an accumulation of IL-17 producing cells that are associated with autoimmunity and inflammatory disease [26]. Other characteristic observations among T cells include decreased numbers and proportions of CD4+ and CD8+ naïve T cells and increased numbers and proportions of late-stage differentiated effector memory CD4+ and CD8+ T cells [7, 8]. These changes are largely driven by a combination of three factors: first, lower numbers of haematopoietic stem cells which also exhibit intrinsic damage and a phenotype skewed towards the myeloid lineage; second, thymic involution and reduced output of antigen-naïve T cells; and third, infection with latent herpes viruses, in particular, *Cytomegalovirus* (CMV) [7, 27]. Among the less abundant T cell populations, natural regulatory T cells (nTREGs) increase with ageing whereas inducible TREGs decrease [28]. It is unclear whether the suppressive capability of these cells is altered with ageing: an increase could promote immune suppression and cancer, whereas a decrease could promote immune activation and autoimmunity [28]. T cells that express natural killer cell-associated cell-surface proteins (NKT-like cells) increase with ageing and exhibit similar changes to their phenotype, functional properties, and specificities as with the broader populations of CD4+ and CD8+ T cells [22]. With ageing, there is a decline in the number of *γδ* T cells; however, age per se, in the absence of chronic infections, is associated with a decline in V*δ*2 cells (60–80% of *γδ* T cells), whereas V*δ*1 (15–20% *γδ* T cells) remain stable [29]. As with T cells, ageing is associated with a decline in the numbers and proportions of naïve B cells, an accumulation of memory B cells with limited specificities, and less robust antibody production by plasma cells in response to novel antigens [30]. Some of these parameters, when clustered together, have been shown to predict all-cause mortality in studies of octogenarians and nonagenarians [31, 32]. These characteristics, which were referred to as the Immune Risk Profile (IRP), included low numbers and proportions of B cells, high numbers and proportions of late-stage differentiated CD8+ T cells, poor T cell proliferation in response to mitogens, and a ratio of CD4+ T cells to CD8+ T cells less than 1.0 [31, 32]. Infection with CMV was part of the IRP [33], and later work showed that plasma IL-6 was associated with frailty, cognitive decline, and mortality [34, 35].

Many features of an ageing immune system appear to be driven by CMV, and infection with this virus has been linked to poor vaccine responses in both young and elderly individuals [36–39]. The effects of CMV on the effectiveness of vaccination remains a contentious issue, however, with some studies showing no effect in the elderly [38, 40, 41] and others even showing vaccine-boosting effects of CMV in young individuals [42]. Irrespective of CMV serostatus, elderly individuals, exhibiting other established hallmarks of an ageing immune system, generally mount less robust immune responses to vaccines compared to young individuals [43]. Aside from influencing responses to novel antigens, CMV infection has also been directly associated with frailty [44] and cognitive impairment [45], and some studies have shown that infection results in earlier mortality [46–48].

## 3. Anti-Immunosenescence Effects of Exercise: General Observations

The idea that aerobic exercise training could influence immunosenescence developed in the late 1990s, almost two decades after the field of exercise immunology, was established [49]. Many studies have investigated how the immune system responds acutely (i.e., over minutes, hours, and days) to different modes, durations, and intensities of single and repeated exercise bouts. Generally, exercise bouts are considered “immunostimulatory” partly due to the rapid exercise-induced leukocytosis that was observed in early studies [50–52]. Among lymphocytes, for example, and in particular T cells and natural killer cells, subpopulations that exhibit strong effector function rapidly and substantially increase in blood during exercise [1, 53, 54]. Subsequently, in the hours following exercise, the same effector cells migrate to tissues, leaving blood to search for cells infected with viruses, bacteria, or those that have undergone a malignant transformation [55, 56]. It is likely that these principles also translate to other cells of the innate immune system, such as monocytes and neutrophils, but these cells exhibit different exercise-induced kinetics compared to lymphocytes. Considering that these processes might reduce the risk of infections [57] and facilitate the detection and elimination of tumours [58], then these exercise-induced changes could be interpreted as countering some aspects of immunosenescence. In addition to the short-term “immunostimulatory” effects of exercise bouts, long-term aerobic exercise training interventions, in both young and elderly individuals, elicit strong anti-inflammatory effects that might counter inflammageing [6]. It seems that these exercise-induced effects may not have their roots in immune cells though and may be due to other cytokine producing cells (e.g., myocytes, adipocytes, fibroblasts, endothelial, and epithelial cells) considering that long-term changes to the function and phenotype of most immune cells are not brought about by regular exercise training [6, 10].

Other studies have examined basal or resting immune function in participants of cross-sectional or longitudinal studies, categorising individuals as being sedentary, inactive, physically active, or extremely active using self-report questionnaires. A more recent approach has been objectively measuring the volume and intensity of free-living activities using wearable activity monitors. However, the data generated by these devices is complicated to interpret using globally recommended physical activity guidelines (e.g., 150 minutes of moderate-to-vigorous intensity physical activity each week *on top* of normal activities of daily living) [59–61]. Wearable activity monitors capture activity accumulated both purposefully (e.g., through exercise) and through daily living, but because they do not discriminate between the two, it has been suggested that a total of ~1000 minutes of moderate-to-vigorous intensity activity per week is a more appropriate target to assess with these devices [62]. Despite these important nuances, regularly active individuals appear to exhibit better overall immune function than inactive individuals, even in the elderly [10]. For example, one of the most robust tests of immune competence is the antibody or cell-mediated response to novel antigens, often administered experimentally by vaccination. Both humoral and cell-mediated responses to vaccination appear to be stronger in active compared to inactive individuals and those who have engaged in structured exercise in the months leading up to vaccine administration [5, 63]. Indeed, different forms of exercise appear to amplify vaccine responses, facilitating long-term immunity in the months after vaccination [5, 63]. These effects have been shown by studies that have not controlled for the degree of immunological ageing (e.g., stratifying by CMV serostatus) suggesting powerful effects of exercise.

Although many aspects of immune function change with ageing, and some of these alterations can be bolstered or restored transiently by different forms of exercise, it remains to be determined whether clinically meaningful and long-term exercise-induced changes to the established hallmarks of immunosenescence can be observed. However, evidence is beginning to accumulate showing that some of these parameters are at least sensitive to change by aerobic exercise training. In addition, theories have been put forward for mechanisms that might underlie possible anti-immunosenescence effects of exercise. So far, this work has largely focused on an ageing adaptive immune system with an emphasis on T cells.

## 4. Anti-Immunosenescence Effects of Exercise: Proposed Mechanisms

It has been suggested that aerobic exercise training might prevent or delay immunological ageing by limiting the expansion of senescent late-stage differentiated T cells, which is a hallmark of immunosenescence [2, 3]. In this hypothesis, it is proposed that three exercise-induced processes bring about this effect. First, cells of a late-stage differentiated phenotype are mobilised into peripheral blood during single bouts of exercise. Second, these cells extravasate from blood, homing to peripheral tissues, where they are exposed to proapoptotic stimuli (e.g., reactive oxygen species, glucocorticoids, and cytokines). Third, assuming that the “size” of the adaptive immune system is fixed, the naïve T cell repertoire expands in response to the immunological “space” that has been created, initiated by a hypothetical negative feedback loop that governs the number of naïve and memory cells and perhaps bolstered by exercise-induced thymopoiesis and/or extrathymic T cell development in tissues such as the liver.

Several parts of this hypothesis are supported by robust findings in exercise immunology. For example, many investigations have shown that late-stage differentiated T cells are mobilised into peripheral blood during bouts of moderate- and vigorous-intensity aerobic exercise, followed by an assumed, in humans at least, extravasation to peripheral tissues [53, 54]. In addition, there is evidence from studies in mice that lymphocyte apoptosis occurs postexercise in tissues that are thought to be the homing destination of mobilised cells [64]. This process occurs in parallel with an increase of haematopoietic stem cells in the bloodstream and at these homing sites, which seems to be an effect of apoptotic cells and cell debris [65]. Such apoptosis-induced haematopoietic stem cell mobilisation might result in trafficking to the thymus (or potentially extrathymic sites) stimulating the development of naïve T cells [66]. Finally, contracting skeletal muscle secretes IL-7 [67] which might increase thymic mass and promote the emergence of recent thymic emigrants [68]. Recent studies have provided insight into whether the numbers and proportions of late-stage differentiated T cells in peripheral blood are sensitive to change with long-term exposure to aerobic exercise training. These studies have been discussed in detail elsewhere [4] but are summarised below.

## 5. The Effects of Aerobic Exercise Training on Late-Stage Differentiated T Cells

There are no randomised and controlled trials that have examined whether aerobic exercise training reduces the numbers and proportions of late-stage differentiated T cells resulting in clinically meaningful changes to overall immune function. However, several cross-sectional studies have provided initial evidence that the characteristics of the T cell pool vary between individuals who have been exposed to different volumes and intensities of aerobic exercise training over periods of their life. In addition, limited evidence suggests that the characteristics of the T cell pool are sensitive to modulation over several months when the volume and intensity of aerobic exercise training are changed.

To summarise, it has been shown that individuals with a high cardiorespiratory fitness, which largely reflects an active lifestyle, exhibit lower proportions of late-stage differentiated T cells and higher proportions of naïve T cells in resting blood samples [69]. The effects of cardiorespiratory fitness appeared to be largely independent of age, body composition, and CMV serostatus [69]. Similar observations have been made in young adults who engage in structured exercise that is typical of training for competitive team sports [70, 71]. In the elderly, however, an active lifestyle only appears to limit the accumulation of late-stage differentiated T cells and does not appear to substantially affect the proportions of naïve T cells [72]. Thus, these findings largely support an anti-immunosenescence effect of aerobic exercise training, particularly in young adults. However, individuals undertaking a much higher volume of endurance training appear to exhibit exaggerated signs of T cell immunosenescence compared to less active age-matched controls [73, 74]. Examples of these changes include a greater accumulation of late-stage differentiated T cells, fewer naïve T cells, and reduced thymic output, as shown by lower levels of T cell receptor rearrangement excision circles in resting blood samples [73, 74]. These effects appear to be most prominent in younger compared to older athletes [74]. Thus, these findings could be interpreted as being a proimmunosenescence effect of very prolonged and repeated aerobic exercise bouts. In support, it has been shown that over six months, when very prolonged endurance training was undertaken by both younger and older athletes, the numbers and proportions of late-stage differentiated T cells increase and the numbers and proportions of naïve T cells decrease [75, 76].

To conclude, it seems likely that individuals who meet or exceed by less than approximately five times, the recommendations for physical activity (i.e., accumulating 150 minutes of moderate intensity or 75 minutes of vigorous-intensity activity on a weekly basis) exhibit less marked immunosenescence than age-matched controls who are not active [4]. However, individuals who take part in regular very prolonged or extreme aerobic exercise over their lifetime may exhibit changes in the T cell pool that are indicative of exaggerated immunosenescence [4]. It seems that both the anti-immunosenescence effects of typical aerobic exercise training and the proimmunosenescence effects of extremely prolonged endurance training are most prominent in young individuals. This indicates that the magnitude of effect brought about by aerobic exercise training is relatively modest, or the effects are at least masked within individuals who already exhibit an aged immune profile. Thus, the limited evidence to date suggests that anti-immunosenescence effects of aerobic exercise training might have most utility in delaying the onset of immunological ageing, rather than restoring aged immune profiles. Although there is some evidence for an anti-immunosenescence effect of aerobic exercise training on late-stage differentiated T cells, some concepts that underlie the possible biological mechanisms are debated. Indeed, many questions remain unanswered, such as how many late-stage differentiated T cells truly classify as being senescent, whether these cells can and should be removed, and whether the “size” of the immune system is fixed. These themes are summarised below with a commentary on whether some anti-immunosenescence effects of aerobic exercise training could be brought about indirectly.

## 6. What Are the Most Likely and Desirable Anti-Immunosenescence Effects of Exercise?

A theme of the original idea for how aerobic exercise training might counter immunosenescence has been the removal of senescent late-stage differentiated T cells [2, 3]. This process is considered desirable for two primary reasons: first, because it is thought some of these cells are unable to function and second, because these senescent cells are thought to be a waste of “space” assuming the “size” of the immune system is fixed.

The concept of a finite amount of immunological space came about, in part, because it has traditionally been assumed that thymic output of naïve T cells is negligible after adolescence [14]. This implies that there is an upper limit to the number of T cells in the immune system and a fixed number of naïve cells capable of mounting responses to novel antigens [14]. Thus, it has been proposed that antigen-naïve cells could be “used up” due to ongoing differentiation into memory cells that “fill up” immunological “space” [14]. In addition, it has been suggested that the accumulation of virus-specific T cells over a lifetime may lead to a “squeezing out” of T cells targeting less dominant viruses or nonpersistent infections, potentially leading to loss of viral control [77]. Although studies continue to support the age-associated reduction in thymic output, evidence points towards a gradual decline, whereby thymic function persists, albeit reduced, up until around 70 years of age [78]. Moreover, the concept of a fixed amount of immunological space has been strongly debated [79, 80]. For example, CMV-seronegative recipients of a CMV-infected kidney demonstrate an “enlargement” of the CD8+ T cell compartment, shown by an appearance and expansion of CMV-specific T cells, but a maintenance (and not deletion) of preexisting Epstein Barr virus-specific and influenza-specific T cells over time [79]. Although it remains up for debate if there is a limit to the “size” of the immune system and the number of naïve T cells within in it, the assumption that low numbers of naïve cells increase the susceptibility to infection has not been tested experimentally [8]. However, a removal of late-stage differentiated cells may be desirable for other reasons, as for example, these cells might contribute to the age-associated increase in inflammatory activity [14].

Assuming late-stage differentiated T cells remain functional, it is likely that they have an important role in controlling latent viruses such as CMV. These cells, which do not express CD27, CD28, CD62L, and CCR7, but express CD45RA, CD57, and KLRG1 [7], almost exclusively accumulate due to CMV infection and reactivation and do not accumulate in elderly CMV-seronegative individuals [7, 81, 82]. Thus, a targeted removal of these cells by exercise is probably only relevant to individuals infected with CMV, but this is a large proportion of the worldwide population (30–90% depending on age, ethnicity, socioeconomic status, and geographical location) [83]. It is however well established that in CMV-seropositive individuals, approximately 10% of the CD4+ and CD8+ T cell pool becomes specific for this virus [84]. Although such large accumulations of CMV-specific T cells are often interpreted to be deleterious, it is unknown what proportion of the T cell pool needs to be specific for CMV to ensure adequate viral control [85]. The importance of fully functioning late-stage differentiated T cells in controlling latent viruses is highlighted by the complications that arise from CMV reactivation and CMV disease in patients receiving solid organ or stem cell transplants, which in part is due to loss or suppression of cell-mediated immunity [86]. Indeed, protocols have been developed to adoptively transfer CMV-specific CD8+ T cells to prevent viral reactivation in transplant recipients [87]. Even in healthy individuals, evidence points towards an important role for CMV-specific immune surveillance. For example, in a longitudinal analysis of elderly individuals, it has been shown that a lower proportion of naïve CD8+ T cells, a higher proportion of memory CD8+ T cells, combined with a robust proinflammatory response to CMV, is associated with survival [88].

Along with uncertainty over how many CMV-specific late-stage differentiated T cells are required for antiviral control, it is unclear what proportion of these cells classify as being truly senescent. The assumption that many of these cells are senescent came about because early studies showed that the proportion of antigen-stimulated IFN-*γ* producing cells among elderly donors was lower than in young donors [89]. However, the cumulative IFN-*γ* production was higher in the elderly due to greater absolute numbers of these cells compared to the young [89], and this could therefore be a mechanism to limit excessive inflammatory activity when controlling CMV in the elderly. Most CMV-specific cells are multifunctional, producing IFN-*γ*, IL-2, and TNF-*α*, and have telomeres of intermediate length; therefore, do not classify as senescent [90]. Although some CMV-specific cells express programmed death-1 (PD-1), this cell-surface protein is thought to better represent exhausted cells that have impaired effector function and are not truly senescent [91, 92].

Although it might be desirable to remove truly senescent late-stage differentiated T cells, it is likely that a biological explanation exists for why these cells accumulate [14]. For example, in vitro experiments have shown that senescent CD8+ T cells, defined as cells being incapable of cell division, are resistant to apoptosis [93]. Conversely, other in vitro work has shown that senescent T cells, defined by expression of cell-surface proteins that have been associated with senescence such as CD57 and KLRG1, are *more* susceptible to H_2_O_2_-induced apoptosis than naïve cells [94, 95]. When examining CMV-specific CD8+ T cells in vitro, it has been shown that rather than being resistant to apoptosis, these cells are equally as susceptible to apoptosis as the broader pool of CD8+ T cells [96]. However, in vivo experiments show that peripheral blood CMV-specific CD8+ T cells exhibit high levels of the antiapoptotic protein Bcl-2, potentially rendering them insensitive to Fas-L/Fas-R-induced death [97]. Currently, it remains unclear whether CMV-specific late-stage differentiated T cells, some of which may classify as being senescent, are resistant to apoptosis in vivo. Thus, it is not certain if these cells would be susceptible to exercise-induced apoptosis as part of countering an ageing immune system, perhaps implicating other indirect mechanisms.

If aerobic exercise training invokes anti-immunosenescence effects by limiting the accumulation of CMV-specific late-stage differentiated T cells, it could be that these changes are brought about indirectly, by limiting viral reactivation, improving redox balance, and countering inflammageing. For example, many individuals who accumulate large volumes of sedentary behaviour and do not engage in physical activity are likely to be overweight or obese, and it is possible that CMV will reactivate frequently because adiposity drives chronic systemic inflammation and oxidative stress [18, 98]. In turn, proinflammatory cytokines and reactive oxygen species reactivate CMV directly [99, 100]. Aerobic exercise training decreases visceral and subcutaneous adipose tissue [101], providing a potent anti-inflammatory stimulus that helps maintain redox balance [6, 102]. Exercise-induced improvements in redox balance may also prevent dysfunction of late-stage differentiated T cells, considering that activation of the p38 mitogen-activated protein kinase signalling pathway by excess reactive oxygen species prevents T cell proliferation [103]. Thus, aerobic exercise training might delay the accumulation of late-stage differentiated CMV-specific T cells by reducing viral reactivation, or preventing T cell dysfunction, and by limiting adipose tissue accumulation and dysfunction that drives inflammation and oxidative stress.

In support of the idea that sedentary behaviour and low levels of physical activity might exacerbate immunosenescence, perhaps via dysregulated adipose tissue, overweight or obese individuals are at a greater risk of viral and bacterial infections, have longer stays in hospital, and exhibit more frequent and prolonged complications, such as antibiotic treatment failure [104–106]. Further, obese individuals exhibit diminished antibody responses to vaccination [107–109], impaired lymphocyte proliferation to mitogens [110], and shorter peripheral blood leukocyte telomere length [111]. In addition, it has been shown that obesity is associated with an accumulation of nTREGs and Th2-phenotype cells that is also seen with ageing [112]. Other reports have shown that obese individuals have large expansions of late-stage differentiated *αβ* T cells and *γδ* T cells, with the latter exhibiting impaired antiviral function [113–115]. Thus, aerobic exercise training might counter immunosenescence indirectly by limiting adipose tissue accumulation and dysfunction that appear to exacerbate ageing processes.

In summary, the proposed anti-immunosenescence effects of aerobic exercise training that focus on limiting the age-associated expansion of late-stage differentiated T cells are desirable based on several assumptions. First, some of these cells are classified as being senescent, failing to adequately control latent viruses, but meanwhile contributing to inflammageing. Second, the “size” of the immune system is fixed and the capacity to produce antigen-naïve T cells is limited, and this process increases the risk of infections and cancer in the elderly.

## 7. Immunosenescence and Cancer: An Overview

In 2015, there were 17.5 million cases of cancer worldwide causing 8.7 million deaths, implicating malignancies as the second most common cause of mortality behind cardiovascular disease [116]. The incidence of cancer is increasing worldwide, as shown by a 33% rise in cases between 2005 and 2015, largely driven by an ageing population [116]. This age-associated increase in cancer incidence occurs in parallel with the age-associated decline in immune function. However, the idea that immunosenescence causes or at least increases susceptibility to cancer is controversial, even when considering the observed relationships between immune function and cancer risk or disease progression. For example, there is a high incidence of cancer in people who are immunosuppressed such as organ transplant recipients [117]. Supporting the concept that a fully functioning immune system hinders the development of cancer, a longitudinal study over 11 years, has shown that individuals with high natural killer cell cytotoxicity, measured in cells isolated from peripheral blood, exhibit a lower incidence of cancer than individuals with less cytotoxic natural killer cells [118]. Indeed, clinical reports of “spontaneous” cancer regression, “disappearance” of tumours, and patients living with “dormant” cancer for up to 20 years provide anecdotal evidence for the importance of anticancer immunity [119]. However, strong evidence proving links between the age-associated decline in immune competence and the development or progression of cancer is lacking, despite advances in the scientific understanding of tumour biology, the development of disciplines that focus entirely on the immunology of cancer, and the subsequent growth of cancer therapies that manipulate aspects of immune function.

It is now almost universally accepted that the immune system has a fundamental role in the detection and elimination of malignant cells, although some aspects of immune function have also been linked to tumour progression [117]. Indeed, the relationship between these processes and malignancy is referred to as the cancer-immunity cycle [120] and it is thought that when anticancer immune surveillance becomes impaired, tumour masses become detectable and clinically relevant [117, 121]. Subsequently, research has led to extensive immunological characterisation of tumours, with signature tumour-associated antigens established and prioritised for the development of cutting-edge treatments [122]. Even with conventional cancer therapies, such as chemotherapy and radiotherapy, it is known that the immune system facilitates some of their effects [123]. Some recent cancer therapies stimulate aspects of immune function, for example, by targeting immune check point inhibitory circuits, and many treatments involve administering immune products such as cells, antibodies, or cytokines [124–126]. Finally, some immunological variables predict clinical outcomes in patients with cancer, such as tumour infiltrating leukocytes ([127, 128] this extensive and complex literature will not be discussed). Despite advances in our understanding of the development, detection, and treatment of malignancies, the quest to investigate whether causal or correlational relationships exist between immunosenescence and cancer is hampered by several unanswered and fundamental questions. First, do individuals who go on to develop cancer already exhibit immunosenescent profiles, perhaps due to low grade inflammation and chronic stimulation with viral antigens? Second, does the development of cancer itself cause immunosenescence, perhaps due to tumour-derived inflammation and chronic stimulation with tumour-associated antigens? Third, do processes involved in both cancer and immunosenescence interact to exacerbate immunological decline in patients with malignancies?

In principle, many characteristics of an ageing immune system could contribute towards cancer risk and lead to poor outcomes in patients. For example, chronic inflammation is tumour promoting [13] and the age-associated changes to the cellular composition and functional capability of the immune system might impair the ability to detect and eliminate cancer cells [11, 12, 129]. Candidate examples include defects in the ability of innate immune cells to recognise malignant transformation, such as reduced natural killer cell cytotoxicity or defective toll-like receptor signalling in macrophages and dendritic cells, with the latter exhibiting a reduced ability to effectively activate T cells [11, 12, 129]. Ineffective priming of T cells by dendritic cells may lead to a weakened or anergic T cell response, which might be exacerbated by reduced thymic output of naïve T cells and an accumulation of exhausted T cells that have been chronically stimulated by virus or tumour antigens [11, 12, 129]. Thus, these effects may limit the ability to respond to novel antigens, such as those expressed by malignant cells. Age-associated reductions in less abundant T cell populations, such as *γδ* T cells and CD1d-restricted iNKT cells, may also impair cell-mediated tumour surveillance, and a decline in the number and function of B cells may lead to a less effective antitumour humoral response [11, 12, 129]. Finally, enhanced suppressive mechanisms with ageing may contribute, such as the accumulation of MDSCs, nTREGs, and Th2 cells producing suppressive or inhibitory cytokines such as IL-10 and TGF-*β* [11, 12, 129]. Indeed, some of these inhibitory mechanisms may have their roots within tumour masses, for example, as part of immune evasion strategies adopted by malignant cells or the process of immunoediting, whereby less immunogenic tumour cells are selected for and subsequently persist [130]. Although it seems intuitive that the age-associated decline in immune function could increase cancer risk and hinder cancer therapy, the links between malignancy and biomarkers of immunosenescence remain a debated topic of research. Recent studies examining links between immunosenescence and cancer are summarised in the next section.

## 8. Do Immunosenescent Profiles Increase the Risk of Cancer and Predict Poor Clinical Outcomes in Patients with Malignancies?

If immunosenescence increases the risk of developing cancer, then it might be expected for epidemiological studies to have repeatedly shown relationships between markers of an ageing immune system and the incidence of cancer or cancer-specific mortality. Relationships of this kind are perhaps difficult to detect and interpret however. Only a few characteristics of an ageing immune system have been linked to all-cause mortality, and most studies have either not examined or have failed to detect associations with cancer-specific deaths [31, 33–35, 46–48, 131]. Thus, it is unclear whether classical biomarkers of immunosenescence can estimate the chance of developing cancer or reflect the capacity to detect and eliminate tumour cells. However, other measurements of immune function that have not typically been considered hallmarks of immunosenescence may provide some insight. For example, a broad marker of immune competence, salivary S-IgA, *has* been independently linked to cancer. In a 19-year analysis of mortality data for 639 adults aged 63 years at the time of measurement, there was a significant negative association between the secretion of S-IgA into saliva and all-cause mortality, driven by an underlying association with cancer-specific mortality and in particular with cancers other than lung cancer [132]. Importantly, these relationships withstood adjustment for sex, occupation, smoking, medication use, and self-reported health [132]. Although it is well established that the incidence of cancer increases with age, coinciding with a decline in immune competence, many other factors, including physical activity level, body composition, and diet, which also change with ageing, likely interact. Thus, even when predicting cancer risk with accepted hallmarks of immunosenescence, such as plasma inflammatory activity—a common parameter to have been examined for associations with cancer, the results are likely to be influenced by multiple lifestyle variables and infection history, which have not always been assessed or controlled robustly [133–135]. In the context of inflammation predicting cancer risk, panels assessing multiple biomarkers may have better predictive utility than individual inflammatory variables, but the process of ageing and inflammageing is highly variable between individuals and very closely related to physical functioning [136]. The search for biomarkers that predict cancer risk, cancer-specific mortality, and disease progression in patients has spanned numerous immunological, inflammatory, and genetic variables. It is beyond the scope of this article to provide a thorough discussion of studies examining these general measurements of ageing. Thus, the remainder of this section will summarise relationships between cancer and the most established, robust, and immunosenescence-specific biomarkers.

### 8.1. Infection with CMV and Risk of Developing Cancer

Considering that many characteristics of an ageing immune system are driven by infection with CMV, if immunosenescence can be linked to cancer, it might be expected that CMV-seropositive individuals would demonstrate increased cancer-specific mortality. Links between CMV and mortality are contentious, with some studies reporting that CMV-seropositivity predicts greater all-cause mortality [47, 131] and others showing no effect of infection [44, 137]. In addition, the investigations implicating CMV in mortality mostly demonstrate that this relationship is driven by death from cardiovascular disease rather than by cancer [46, 48, 138]. However, in an analysis of 13,090 immunocompetent individuals aged 40–79 years at recruitment, which showed CMV-seropositivity was associated with increased all-cause mortality over a period of approximately 14 years (age- and sex-adjusted hazard ratio 1.16), cause specific analyses showed that in addition to cardiovascular disease (hazard ratio 1.06), cancer also contributed to the overall associations (hazard ratio 1.13) [139].

A small body of research has attempted to firmly establish links between CMV infection and the risk of developing specific forms of cancer in healthy people. For example, it has been hypothesised that late or recent exposure to CMV might cause breast cancer [140]. This idea was formed on the basis that breast cancer incidence is higher in countries where exposure to CMV occurs later in life, compared to countries where almost the whole population is exposed during childhood [140]. In testing this hypothesis however, there were no differences in CMV serostatus between women who developed breast cancer compared to controls [141]. Yet, in CMV-seropositive women, CMV-specific IgG was higher among breast cancer patients compared to controls, which was interpreted as reflecting recent exposure to CMV [141]. Similarly, it has been shown in CMV-seropositive women that an increase in CMV-specific IgG, measured in serum samples collected at least one year apart and approximately four years prior to breast cancer diagnosis, appears to precede tumour development [142]. However, high or rising CMV-specific IgG could also represent prolonged infection due to exposure early in life, viral reactivation, or superinfection with multiple virus strains [143, 144]. Indeed, associations between the sero-epidemiology of CMV infection and breast cancer incidence are not consistent with measurements of viral DNA in breast tumours [145].

Other literature examining whether CMV increases the risk of cancer has focussed on mortality after organ transplantation. For example, it has been shown in a large study of 22,461 recipients of kidney, heart, liver, or lung transplants that mortality over 10 years, from a variety of causes, was greater when organs from a CMV-seropositive donor were transplanted into a CMV-seronegative recipient [146]. A greater incidence of posttransplantation cancer was observed among CMV-seropositive recipients compared to CMV-seronegative recipients (irrespective of donor CMV serostatus) [146]. Importantly, these associations were lost when controlling statistically for age and sex, challenging the links between CMV infection and cancer risk [146]. Other smaller studies have reported conflicting results. For example, in a study of 455 kidney transplant recipients, it was shown that pretransplant exposure to CMV and posttransplant CMV replication was associated with an increased incidence of cancer [147]. Compared to organ recipients who remained disease free, those who developed cancer also exhibited other signs of immunosenescence, such as large expansions of CD8+CD28− T cells [147]. In contrast, a study of 105 kidney transplant recipients showed that CMV-naïve individuals, compared to recipients who were exposed to CMV pre- or posttransplant, demonstrated a *greater* risk of cancer, which was attributed to antitumour activities of *γδ* T cells that accumulate with CMV infection [148]. The relationships between CMV infection and the risk of developing cancer are unclear, and further research is required. However, it may be that infection with this virus per se does not increase the risk of developing cancer, and rather it is the consequences of infection that are more important. For example, in a study of 117 kidney transplant recipients identified as being at high risk of cutaneous squamous cell carcinoma, the age- or CMV-associated accumulation of CD8+CD57+ T cells was a strong predictor of cancer development, rather than viral infection directly [149].

### 8.2. Infection with CMV and Tumourigenesis

The majority studies examining links between CMV and malignancy have searched for viral DNA, RNA, and proteins in tumour cells from individuals with a confirmed cancer diagnosis or have examined the effects of infecting cancer cell lines with CMV in vitro [150–152]. Most research implicates CMV in tumourigenesis, rather than being a factor that increases the risk of developing cancer, and a common view is that CMV is “oncomodulatory,” infecting tumour cells and modulating their malignant properties [150–152]. Changes include increasing chromosome instability, stimulating proliferation by dysregulation of the cell cycle, enhancing resistance to apoptosis, facilitating invasion, migration and endothelial adhesion, promoting angiogenesis, and contributing to immune evasion [150–152].

The cancer diagnosis to have received most attention in the context of CMV are malignant gliomas—the most common primary brain tumours in adults—comprising both astrocytoma and glioblastoma multiform [152, 153]. It was first established in 2002 that a high percentage of malignant glioma tumours are infected with CMV and multiple gene products are expressed that likely contribute to oncogenesis [154]. These findings have been confirmed by multiple studies [153]. It has also been shown that there is a negative association between the number of tumour cells infected with CMV and length of survival [155]. In addition, treating glioma patients with Valganciclovir, which limits CMV reactivation, improves two-year survival rate (by 72%) extending median survival by approximately 43 months [156]. CMV has subsequently been detected in tumour cells from patients with cervical cancer [157, 158], breast cancer [145, 159], colorectal cancer [160], prostate cancer [161], and gastric cancer [162]. The specific role(s) that CMV might have in oncogenesis and whether this virus negatively influences treatment outcomes is uncertain for most of these cancers, and the direction of effect may even be surprising. For example, despite the complications that CMV can have during the treatment of haematological malignancies, CMV reactivation is associated with a *decreased* chance of cancer relapse in acute myeloid leukaemia [163, 164].

### 8.3. Relationships between Cancer and Cellular Markers of Immunosenescence

In addition to CMV infection, other hallmarks of immunosenescence have been examined in patients diagnosed with different forms of cancer, and comparisons have been made to healthy individuals, or relationships with survival have been explored. For example, compared to healthy controls, patients with glioblastoma multiform exhibit lower percentages of total T cells, an accumulation of *γδ* T cells, and expansions of *αβ* T cells with CD4+CD28− and CD4+CD57+ phenotypes [165]. In addition, higher levels of CD4+CD28− and CD4+CD57+ cells measured three weeks after surgery in these patients were associated with shorter survival [165]. Similar accumulations of CD28− and CD57+ cells have been observed within the CD8+ T cell pool in patients with lung cancer [166, 167]. These expansions of CD8+CD28− cells were linked to broader aspects of immune function and clinical outcomes, such as the efficacy of a therapeutic cancer vaccine, assessed by length of survival following administration [167]. Other immune parameters, such as the frequency of CD57+ NKT-like cells in gastric cancer patients, have been reported as being comparable to healthy controls [168]. However, among the most advanced-stage patients, higher proportions of CD57+ NKT-like cells were associated with shorter survival [168]. Likewise, analysis of PD-1 expression on T cells from patients with acute myeloid leukaemia revealed no differences compared to healthy controls at the time of diagnosis, but PD-1 expression increased substantially at the time of relapse [169]. Thus, it seems that the timing of blood sampling and stage of disease are critical for interpreting these measurements. Other classical hallmarks of immunosenescence have also been examined, with reports of low CD19+ B cell numbers in patients with lung cancer, along with a decreased ratio of CD4+ T cells to CD8+ T cells, which has also been shown in patients with late-stage melanoma [166, 167, 170]. These late-stage melanoma patients also exhibited marked changes to the composition of the *γδ* T cell pool, exhibiting a decline in the number of V*δ*2 cells, no change in V*δ*1 cells, and a subsequent increase in the ratio of V*δ*1 to V*δ*2 cells [170]. A higher frequency of V*δ*1 cells was negatively associated with survival, driven by V*δ*1 cells with an early differentiated phenotype (as with CD8+ early differentiated *αβ* T cells), but V*δ*2 cells were not linked to clinical outcomes [170].

It is possible that the alterations within the T cell pool reported in patients with cancer are treatment- or disease-induced acceleration of normal ageing processes, such as reduced thymic output. For example, it has been shown that patients with breast cancer, compared to healthy controls, exhibit lower levels of T cell receptor rearrangement excision circles and lower percentages of recent thymic emigrants, observed in parallel with fewer CD8+ naïve T cells and shorter telomeres assessed in peripheral blood mononuclear cells [171]. Indeed, studies have reported incremental changes to biomarkers of immunosenescence following repeated administration of chemotherapy, including lower ratios of CD4+ T cells to CD8+ T cells and accumulations of CD28− and CD57+ cells in patients with breast and lung cancer [166, 172]. Both murine and human studies have shown that common chemotherapeutic drugs induce a senescence-associated secretory phenotype in peripheral blood T cells [173–175]. These so-called therapy-induced senescent cells, which accumulate and contribute to local and systemic chronic inflammation, can be identified by elevated expression of *p*16^INK4a^, a tumour suppressor [173–175]. In T cells, *p*16^INK4a^ is a marker of molecular ageing in healthy individuals, and its expression positively correlates with age and physical inactivity [174, 176]. In patients with cancer, T cell *p*16^INK4a^ expression increases in a dose-dependent manner with chemotherapy administration [174, 175]. Interestingly, removal of *p*16^INK4a^ expressing T cells increased physical activity and strength in tumour bearing mice [173]. Considering that in humans, *p*16^INK4a^ T cell expression correlated with self-reported fatigue in the context of breast cancer [173], and that exercise is recommended for its fatigue-countering effects [177, 178], then it might be that some of the exercise-induced benefits for patients with cancer are, in part, brought about by interaction with *p*16^INK4a^. Moreover, removal of *p*16^INK4a^ expressing T cells in mice has been shown to reduce cancer recurrence [173]. Thus, *p*16^INK4a^ seems to be an important marker of T cell senescence that not only interacts with exercise but might also be linked with survival, perhaps due to better anticancer immunity. In support, it has been demonstrated that IL-15 enhances the activity of tumour-specific T cells via delaying or reversing senescence, as shown by lower expression of *p*16^INK4a^ among other markers of senescence [179]. Indeed, recent research has indicated that the capacity of peripheral blood T cells to recognise and respond to tumour-associated antigens is a predictor of survival in several malignancies, including breast cancer [180, 181], colon cancer [182], melanoma [183, 184], and hepatocellular carcinoma [185].

## 9. Does Exercise Elicit Anticancer Effects by Countering Immunosenescence?

### 9.1. Relationships between Active Lifestyles and Cancer: An Overview

An active lifestyle reduces the risk of developing cancer. In an analysis of self-report leisure-time physical activity data from 661,137 people in six population-based prospective cohorts, it was shown that the most active individuals (who performed more than ten times the recommended minimum volume of exercise each week) had a 31% lower cancer mortality risk compared to individuals reporting no activity [186]. Further, an investigation of risk for different cancer types among 1.44 million people from twelve prospective studies showed, by comparing individuals in the 90th and 10th percentiles for self-reported leisure-time physical activity, that very active lifestyles are associated with a lower risk of thirteen cancers, including oesophageal adenocarcinoma, liver, lung, kidney, gastric cardia, endometrial, myeloid leukaemia, myeloma, colon, head and neck, rectal, bladder, and breast [187]. The reduction in risk ranged from 10 to 42% among the different cancers, and most associations remained significant after statistical adjustment for body mass index and smoking [187].

Despite substantial reductions in cancer risk brought about by active lifestyles, the relevance and magnitude of effect for external and potentially modifiable factors influencing the development of malignancies has been debated. For example, comparing the total number of tissue-specific lifetime stem cell divisions and the lifetime risk of cancer in the same tissue has indicated that around 30% of the variation in cancer risk is due to external or inherited predispositions [188]. However, an alternative and more extensive analysis of similar data proposed that external factors contribute 70–90% of lifetime cancer risk [189]. A more conservative estimate is provided by investigations of risk factor exposure and cancer incidence. For example, approximately 43% of cancers occurring in the UK in 2010 have been attributed to suboptimal or past exposure to potentially modifiable external factors [190]. Although the most substantial cancer risk factor was tobacco, attributable to 19.4% of all diagnoses, lifestyle factors that can in principle be modified by intervention accounted for a substantial number of cases [190]. Factors included suboptimal diet (9.2%), overweight and obesity (5.5%), consumption of alcohol (4.0%), and inadequate exercise (1.0%) [190]. Other exposures implicated in the development of cancer, although potentially modifiable, are more difficult to avoid for practical, political, and societal reasons (e.g., occupation 10.0%, ultraviolet radiation 3.5%, ionizing radiation 1.8%, infections 3.1%, and reproductive factors 0.9%) [190].

Although current evidence implicates inadequate physical activity as accounting for a modest proportion of cancer diagnoses, these estimates are derived from self-reported physical activity data and the relationships that had previously been established between specific cancers and active lifestyles. Traditionally, research has linked inactive lifestyles with risk of breast, colon, and prostate cancer, with some evidence for endometrial and lung cancer [191]. However, more recent research suggests that a much larger range of cancers are linked to inadequate physical activity, and it seems likely that this list will grow as more data become available [187]. Thus, as new evidence accumulates using objective measurements of physical activity in combination with measurements of body composition that are more sensitive than body mass index, the number of cancer cases attributable to inadequate physical activity may increase. Even if with future research, the fraction of cancers thought to be attributable to inactive lifestyles does not change; this estimate should not be discounted. Preventing just 1% of the 17.5 million cases of cancer worldwide is attractive and further justified by the increasing cancer incidence due to an expanded ageing population [116].

It should be emphasised that the relationships between cancer and exercise or physical activity are not limited to reducing the risk of developing malignancies in healthy individuals. For example, there is a large body of evidence demonstrating that aerobic exercise training can benefit patients with a cancer diagnosis. It is recommended that patients at all stages of the cancer survivorship continuum (e.g., from diagnosis, during and following treatment, to end of life) adhere to the same physical activity guidelines as for healthy individuals, so long as the mode, duration, intensity, and frequency of each exercise session are appropriate for the stage of disease and nature of treatment [177]. This recommendation has been made on the basis that exercise brings about many benefits for patients, including improvements in overall quality of life, cardiorespiratory fitness, strength, flexibility, mood, anxiety, and self-esteem [192, 193]. In addition, aerobic exercise training is an effective method for countering some of the side-effects of cancer treatment, such as fatigue [178]. Most importantly, there are strong relationships between cardiorespiratory fitness or habitual engagement in physical activity with positive treatment outcomes, including reduced disease recurrence and longer survival [192, 193]. These relationships have been demonstrated by observational studies of patients with different malignancies, including breast, prostate, colorectal, and lung cancer [194–198]. In support, the relationship between survival and aerobic exercise training during cancer therapy has been shown by a randomised and controlled trial in patients with breast cancer [199].

### 9.2. Relationships between Active Lifestyles and Cancer: Possible Mechanisms

The mechanisms underlying relationships between regular engagement in physical activity and the risk of developing cancer, some of which are also likely to be relevant to the positive effects that an active lifestyle can have on clinical outcomes in patients, have not been confirmed. This is despite conceptual understanding of the natural mechanisms protecting against cancer, which can be divided into two broad categories: first, defences that reduce or limit exposure to factors that promote malignant transformation and tumour growth and second, defences that target malignant cells more directly [200]. The first category of defences might be considered nonimmune mechanisms and include dietary anticarcinogenic substances, such as antioxidant vitamins; enzymes that actively remove carcinogenic substances, such as antioxidant enzymes; sensors of DNA damage that trigger repair or apoptosis; tumour suppressor genes; telomeres that limit cell division; and inhibitors of angiogenesis, cell migration, or invasion [200]. The second category of defences might be considered immune mechanisms and comprise almost all aspects of humoral and cell-mediated immunity, spanning the innate and adaptive immune compartments [200]. Thus, active lifestyles or aerobic exercise training could conceivably elicit protective effects via interaction with several of these defences [201].

Many of the exercise-induced anticancer mechanisms proposed in the literature have focussed on processes relevant to the cancers traditionally linked with reduced risk brought about by active lifestyles. For example, in the context of colon cancer, it has been suggested that regular aerobic exercise training or physical activity increases gastrointestinal transit time, reducing mucosal exposure to potentially carcinogenic substances [191]. With breast cancer, it has been proposed that regular aerobic exercise training or physical activity reduces lifetime exposure to oestrogen, perhaps due to delayed menarche and reduced ovulatory cycles [191]. Finally, the traditionally observed lower risk of prostate cancer has been attributed to an exercise-induced production of sex hormone binding globulin that reduces exposure to testosterone [191]. Other more widely applicable and commonly proposed anticancer effects of regular aerobic exercise training or physical activity include reducing oxidative stress and inflammation, limiting adipose tissue accumulation, modulating the insulin-like growth factor axis, and stimulating immune function [201, 202]. Most research has sought to identify mechanisms that could explain the lower incidence of cancer in very active individuals. However, limited research has investigated mechanisms underlying better clinical outcomes exhibited by patients who remain regularly active or engaged in structured aerobic exercise training during cancer treatment. For example, a murine model of breast cancer showed that regular wheel running improved the effectiveness of chemotherapy by promoting tumour vascularity, reducing tumour hypoxia, and subsequently enhancing tumour perfusion [203]. A factor that is likely to be relevant to both reducing the risk of developing cancer in healthy individuals, but also improving treatment outcomes in patients, is an exercise-induced enhancement of immune surveillance that might facilitate the detection and elimination of malignant cells. Research exploring this idea has so far focussed on natural killer cells. For example, in humans, it has been shown that an acute bout of vigorous-intensity exercise mobilises highly cytotoxic natural killer cells improving their ability to lyse multiple myeloma and lymphoma cell lines [204]. In addition, murine models of melanoma, lung, and liver cancer have shown that repeated bouts of wheel running stimulate an adrenaline- and IL-6-dependent mobilisation of natural killer cells into peripheral blood, which subsequently extravasate and home to implanted tumours, limiting cancer growth [58]. Indeed, it is likely that research investigating exercise, muscle, tumour, and immune cross-talk, exploring a role for both cellular and soluble mediators, will bring further insights into the most likely anticancer effects of aerobic exercise training [205].

### 9.3. Relationships between Active Lifestyles and Cancer: An Anti-Immunosenescence Effect of Exercise?

Considering that, first, physical activity reduces the risk of developing cancer, second, cancer risk might be modulated by immunosenescence, and third, aerobic exercise training may bring about anti-immunosenescence effects, it has never been investigated whether the anticancer properties of exercise are elicited by countering immunological ageing. To emphasise, as healthy individuals who undertake a moderate volume of aerobic exercise throughout their lifetime appear to have a reduced risk of developing cancer and may exhibit less marked immunosenescence than those who are inactive, it could be hypothesised that these two observations are linked. In other words, does aerobic exercise training reduce the risk of developing cancer by limiting the age- and infection-associated accumulation of late-stage differentiated T cells along with concomitant changes in the naïve T cell pool, and perhaps overall immune function? Although worthy of investigation, this hypothesis may not be confirmed. First, in healthy individuals who are not immunosuppressed, the characteristics of the T cell pool have not been directly and robustly linked to the development of cancer. Second, although individuals undertaking extremely large volumes of endurance exercise may exhibit exaggerated signs of immunosenescence, there is generally a dose-response relationship between exercise and all-cause mortality and cancer-specific mortality [186, 187]. Despite the exception of links between ultraendurance exercise and cardiovascular risk [206], engagement in very high-volume exercise has not been linked with morbidity and mortality from other diseases, especially cancer [186, 187]. However, anti-immunosenescence effects of aerobic exercise training might have more relevance for patients who have been diagnosed with cancer and are undergoing treatment. As outlined earlier, higher cardiorespiratory fitness and regular exercise is linked to *successful* cancer treatment and *longer* survival [192–199]. In addition, exacerbated immunological decline, assessed by measuring cellular markers of immunosenescence, is linked to *unsuccessful* cancer treatment and *shorter* survival [165–168, 170]. Thus, it is possible that positive treatment outcomes, exhibited by patients with high cardiorespiratory fitness, who are physically active or undertake regular aerobic exercise training and exhibit less marked immunosenescent profiles compared to their inactive counterparts, could be driven by an anti-immunosenescence effect of exercise.

## 10. Conclusions

Considering that ageing results in impairments to innate and adaptive immunity and that a single bout of exercise is a powerful stimulus of immune function, it is appealing that regular aerobic exercise training might exert anti-immunosenescence effects. Evidence is accumulating in support of this idea, and these exercise-induced effects might delay the age-associated alterations to immune function. Although it is unknown whether such effects are brought about by exercise directly, such as a targeted removal of dysfunctional T cells, or indirectly, such as lower inflammatory activity and less frequent viral reactivation, it is conceivable that these changes will bring about considerable benefits to health, including reduced morbidity and mortality from infectious disease and cancer.

The links between biomarkers of immunosenescence and the risk of developing cancer in healthy individuals remain to be fully established. However, evidence is beginning to show that patients diagnosed with cancer exhibit immunosenescent profiles. Analysis of disease outcomes, in combination with measurements estimating the extent of immunosenescence, suggests that exacerbated immunological ageing is linked to unsuccessful clinical outcomes in several cancers. Considering that active lifestyles reduce the risk of developing cancer and are associated with positive treatment outcomes in patients, it is possible that some of these effects could be driven by an anti-immunosenescence effect of regular aerobic exercise training.
